# Development of a Deep-Learning Algorithm for Small Bowel-Lesion Detection and a Study of the Improvement in the False-Positive Rate

**DOI:** 10.3390/jcm11133682

**Published:** 2022-06-26

**Authors:** Naoki Hosoe, Tomofumi Horie, Anna Tojo, Hinako Sakurai, Yukie Hayashi, Kenji Jose-Luis Limpias Kamiya, Tomohisa Sujino, Kaoru Takabayashi, Haruhiko Ogata, Takanori Kanai

**Affiliations:** 1 Center for Diagnostic and Therapeutic Endoscopy, Keio University School of Medicine, 35 Shinanomachi, Shinjuku, Tokyo 160-8582, Japan; sujino_t@hotmail.co.jp (T.S.); kaoru0902@yahoo.co.jp (K.T.); hogata@z8.keio.jp (H.O.); 2 Division of Gastroenterology and Hepatology, Department of Internal Medicine, Keio University School of Medicine, Tokyo 160-8582, Japan; tmfmhre03@yahoo.co.jp (T.H.); annapanna_live_s_42@yahoo.co.jp (A.T.); sakuraihinako1206@yahoo.co.jp (H.S.); yukie_tennis_0929@yahoo.co.jp (Y.H.); kenjilimpias@gmail.com (K.J.-L.L.K.); takagast@z2.keio.jp (T.K.)

**Keywords:** video-capsule endoscopy, deep learning, obscure gastrointestinal bleeding, angioectasia, tumor

## Abstract

Deep learning has recently been gaining attention as a promising technology to improve the identification of lesions, and deep-learning algorithms for lesion detection have been actively developed in small-bowel capsule endoscopy (SBCE). We developed a detection algorithm for abnormal findings by deep learning (convolutional neural network) the SBCE imaging data of 30 cases with abnormal findings. To enable the detection of a wide variety of abnormal findings, the training data were balanced to include all major findings identified in SBCE (bleeding, angiodysplasia, ulceration, and neoplastic lesions). To reduce the false-positive rate, “findings that may be responsible for hemorrhage” and “findings that may require therapeutic intervention” were extracted from the images of abnormal findings and added to the training dataset. For the performance evaluation, the sensitivity and the specificity were calculated using 271 detectable findings in 35 cases. The sensitivity was calculated using 68,494 images of non-abnormal findings. The sensitivity and specificity were 93.4% and 97.8%, respectively. The average number of images detected by the algorithm as having abnormal findings was 7514. We developed an image-reading support system using deep learning for SBCE and obtained a good detection performance.

## 1. Introduction

Small-bowel capsule endoscopy (SBCE) is an easy and non-invasive procedure that allows observation of the whole small bowel, which is difficult to achieve with conventional endoscopes. However, SBCE takes more than 50,000 images, and reading such a large number of endoscopic images imposes a large burden on physicians. To shorten the reading time and reduce the misreading of lesions, several capsule-endoscopy-reading algorithms have been developed that detect red-colored images [1,2] and reduce the number of capsule-endoscopic images by discarding similar images [3,4] or assist in detecting abnormal candidate images. Since various findings (such as vascular, inflammatory, and neoplastic lesions) are observed in the small bowel, there is a need to create an effective lesion-detection algorithm that has a minimal false-positive rate and can detect a variety of abnormalities with high sensitivity. Deep learning has recently been gaining attention as a promising technology to improve the identification of lesions, and lesion-detection algorithms using deep learning have been developed. For example, lesion-specific algorithms have been created for bleeding [5], hookworm [6], angioectasia [7,8], and mucosal breaks [9,10]. More recently, studies have shown that various lesions in the small bowel can be detected with high sensitivity by deep learning; however, there is no report of the false-positive rate [11,12,13]. Moreover, most developed algorithms use the Medtronic platform [7,8,10,11,13,14,15,16].

In the present study, we developed a lesion-detection algorithm for the Olympus platform, using deep learning in collaboration with Olympus, and evaluated its performance by assessing the sensitivity and the false-positive rate, which are important in achieving a reduction in reading time.

## 2. Materials and Methods

### 2.1. Study Design

We conducted a retrospective study of 172 cases in which SBCE was performed by the EndoCapsule 10 system (Olympus Corp., Tokyo, Japan). We assumed that the lesion-detection algorithm had a mean sensitivity of 90% and followed the t-distribution, and we set the number of cases for the validation so that the lower limit of the 95% confidence interval was more than 80%. According to this calculation, 35 cases were required for the validation dataset. In addition, we estimated that at least 30 cases would be required for the training dataset, based on our experience in the development of a lesion-detection algorithm using deep learning. Informed consent was obtained from all patients enrolled in the study.

**Figure 1:**
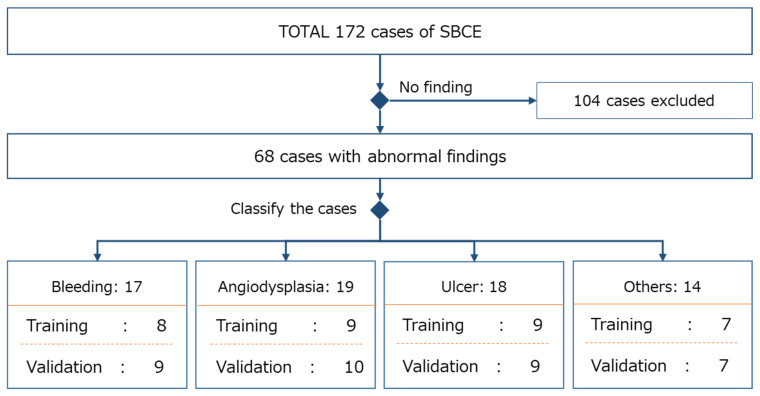
Data selection for the validation and training datasets.

The validation dataset and training dataset comprised images selected from the 172 included cases and were classified so that the findings were equally represented in each dataset. To avoid biases in the validation result through the data selection, each case was randomly allocated to either the training or validation dataset, by lesion category. More specifically, the cases with abnormal findings were selected from the 172 cases and were categorized into four lesion categories usually seen in SBCE (bleeding, angiodysplasia, ulcer, and other). Cases within each lesion category were then equally and randomly allocated to either the validation or training dataset. This resulted in 68 cases with abnormal findings that were classified as bleeding (*n* = 17), angiodysplasia (*n* = 19), ulcer (*n* = 18), and other (*n* = 14). Thirty-five cases were assigned to the validation dataset, and the remaining thirty-three cases were assigned to the training dataset. An approximately equal number of cases in each lesion category were allocated to the validation and training datasets. If the number of cases in a lesion category was not divisible by two, the extra case was allocated to the validation dataset (Figure 1).

### 2.2. Training Dataset for the Lesion-Detection Algorithm with Deep Learning

To specify the image data to be used for training, 33 cases assigned to the training dataset were re-read and abnormal findings were detected. If the lesion-detection algorithm is trained using images with a negligible level of minor findings, the sensitivity of the detection of minor abnormal findings will be improved. However, the false-positive rate would likely worsen because slight changes in the endoscopic images would also be detected, even though they were not abnormal. Therefore, to eliminate such drawbacks and minimize the false-positive rate, we defined abnormal findings as a “finding that may be a lesion responsible for bleeding” and a “finding that requires therapeutic intervention”. Subsequently, three cases in the “other” lesion category were excluded from the training dataset after re-reading.

The resultant training dataset consisted of 49,180 images with abnormal findings and 30,012 images without abnormal findings. The training dataset included 18 images of bleeding, 70 of vascular lesions, 133 of ulcerative lesions, and 35 of neoplastic/other lesions. A total of 256 findings were used for the training dataset.

### 2.3. Lesion-Detection Algorithm with Deep Learning

**Figure 2:**
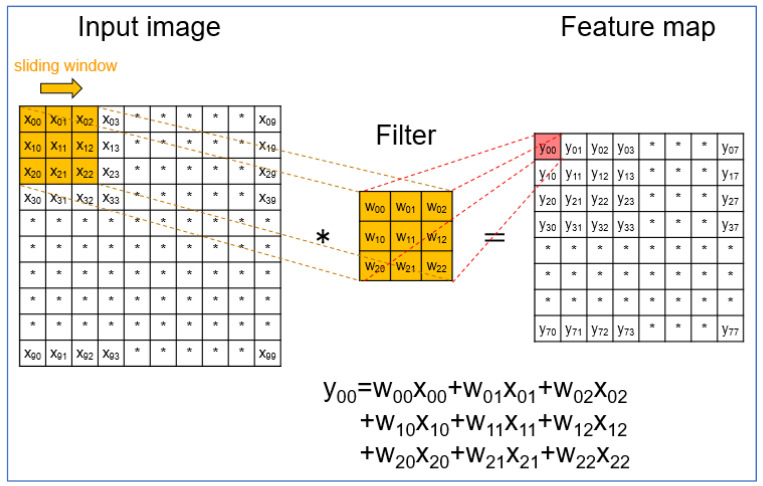
Layout of the first-convolution layer. In the first-convolution layer, each pixel value of the input images (X_00_, X_01_, X_02_, etc.) was translated to the feature maps (Y_00_, Y_01_, Y_02_, etc.) by multiplying the filter weights (W_00_, W_01_, W_02_, etc.) and sliding the filter over the input images (e.g., Y = W_00_X_00_ + W_01_X_01_ + W_02_X_02_ + W_10_X_10_ + W_11_X_11_ + W_12_X_12_ + W_20_X_20_ + W_21_X_21_ + W_22_X_22_).

Recently, deep learning has shown a high level of performance for various image-recognition tasks. In clinical research, deep learning has spread rapidly, and the convolutional neural network (CNN) is most-used. The CNN is a very popular algorithm for image classification and typically comprises convolution layers, activation-function layers, and max-pooling layers. In the first convolution layer, each pixel value of the input images was translated to the feature maps by multiplying the filter weights and sliding the filter over the input images (Figure 2). After the first convolution layer, the subsequent convolution layers were inputted into the feature map of the previous layer instead of the input image.

In the algorithm we developed, ERFNet is used to automatically detect lesion images. The CNN of ERFnet has a novel layer that uses residual connections and factorized convolutions in order to remain highly efficient while still retaining remarkable performance. ERFNet is a deep network with hundreds of layers. The architecture and parameters such as the number of layers, the channels, and the input/output size of each layer are used without any particular modifications [17]. Using ERFNet, segmentation is performed on capsule-endoscopic images to assign a lesion label or a non-lesion label to all pixels in each image. To minimize the influence of misidentified pixels, after segmentation, if there are more than a threshold number of lesion-label pixels in the image, the image is judged as a lesion image.

### 2.4. Validation

The primary evaluation points were the sensitivity, specificity, and number of images detected by the CNN algorithm as having abnormal findings. We considered that both the specificity and the number of detected images (images detected by the algorithm as having abnormal findings) were significant indicators to evaluate the effect of the decreased false-positive rate in terms of reducing the burden of reading.

Regarding specificity, to confirm the effect of the decreased false-positive rate, normal images were identified from the validation dataset. The criterion for detecting normal images was defined as “images without either an abnormality or minor finding such as small red spot or small lymphoid follicles”. Specificity was calculated based on the number of images detected by the CNN algorithm as a percentage of the total number of normal images that were identified. Although SBCE cases involve many images taken throughout the gastrointestinal tract other than the small bowel, the present study focused on small-bowel evaluation. Hence, the number of images detected by the CNN algorithm was counted within the small-bowel section predefined in the validation dataset, and the detection rate was calculated by dividing the number of detected images by the average number of images taken in the small-bowel section.

In some patients with overt bleeding, blood covers most of the images taken after the origin of bleeding. We assumed that the reader would not usually observe these images in detail when reading such cases and that the number of detected images would not be an appropriate indicator of the burden of the reading. Therefore, we also evaluated the average number of detected images and the detection rate in the small bowel of only the cases without massive bleeding.

Sensitivity was calculated based on the agreement rate between the images with abnormal findings identified in the validation dataset and the detection result of the CNN algorithm. The criteria for identifying abnormal findings were the same as for the training dataset, as the detection targets of the lesion-detection algorithm were the images with abnormal findings that directly contributed to the diagnosis.

## 3. Results

For the images in the validation dataset, the mean patient age was 69.3 years, and there were 22 men and 13 women (Table 1). The major indication for SCBE was obscure gastrointestinal bleeding, which accounted for 57% (20 cases), followed by anemia (14%). A total of 271 findings were identified, consisting of 30 findings of bleeding, 46 of angiodysplasia, 153 of ulcers/erosions, and 42 of neoplastic lesions, including polyps and lymphoma (Table 1). The total number of images without abnormal findings in the validation dataset was 68,494.

Regarding the performance of the algorithm, the specificity was 97.8%, and the average number of images in the small-bowel section detected as having abnormal findings was 7514 per case. Among the 31 cases without massive bleeding, the average number of detected images was 3576 per case. The average-detection rate (i.e., the number of detected images divided by the average number of images taken in the small-bowel section) was 16.3% for the total 35 cases and 7.8% for the 31 cases without massive bleeding.

**Figure 3:**
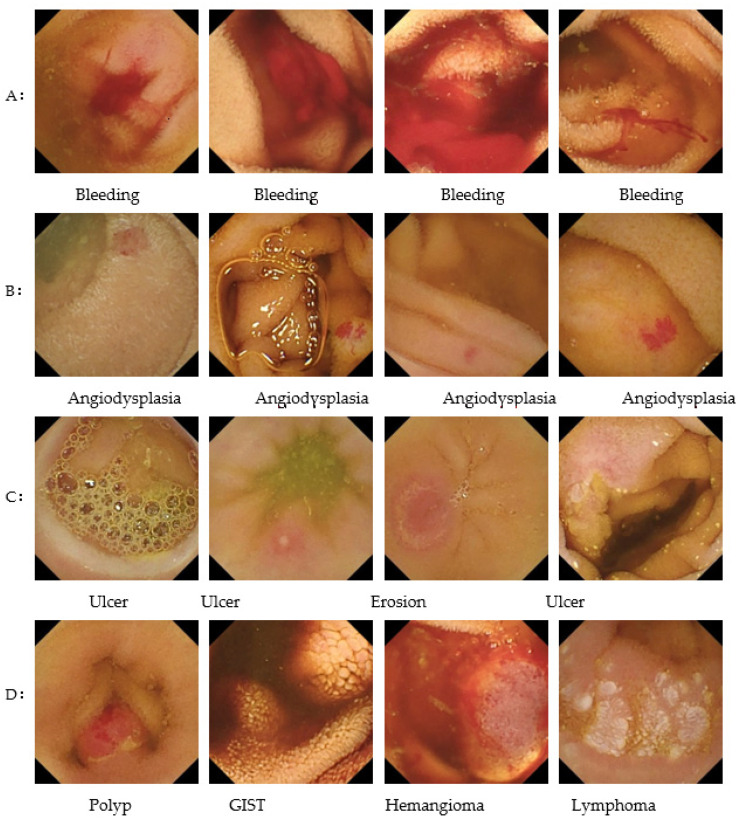
Representative true-positive images correctly detected by the CNN algorithm as showing (**A**) bleeding, (**B**) angiodysplasia, (**C**) ulceration, (**D**) other abnormal findings.

**Figure 4:**
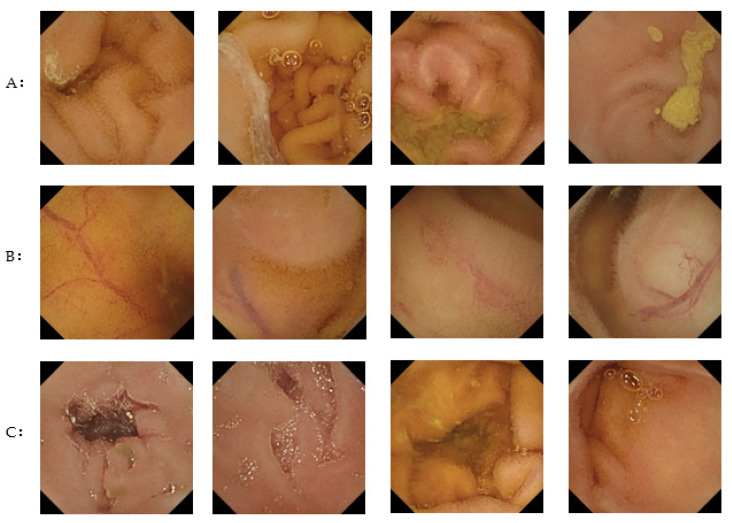
Representative false-positive images detected by the CNN algorithm among the normal image group. (**A**) White debris mistakenly identified as an ulcer, (**B**) normal blood vessel mistakenly identified as angiodysplasia, (**C**) dark part of a surface depression mistakenly identified as angiodysplasia.

**Figure 5:**
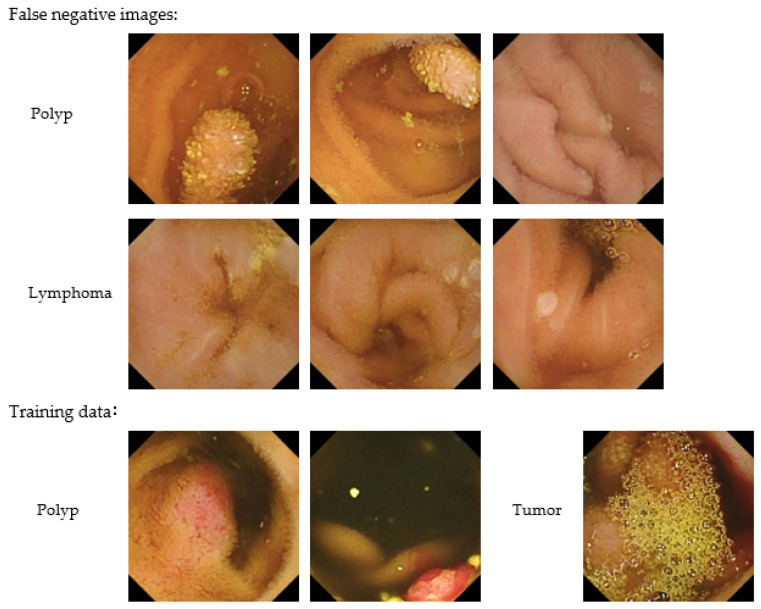
Representative false-negative images and training data for neoplastic lesions.

The sensitivity for each finding was 100% (30/30) for bleeding, 100% (46/46) for angiodysplasia, 92.7% (102/110) for ulceration, 93.0% (40/43) for erosion, 83.3% (10/12) for polyps, and 57.7% (15/26) for lymphoma (Table 2). Figure 3 shows examples of images correctly detected by the CNN algorithm (true-positive images), while Figure 4 shows images incorrectly detected as having abnormal findings (false-positive images). Representative false-negative images and the training data for the neoplastic lesions are shown in Figure 5. All polyp images missed by the current algorithm were white polyps (upper left, Peutz-Jeghers syndrome; upper middle, Peutz-Jeghers syndrome; upper right, FAP). All of the malignant-lymphoma images missed by the developed algorithm in this study were follicular lymphomas with white-lymph follicle-like bumps. In contrast, the polyp training data were red polyps with pyogenic granuloma. The tumor image in the training data used in this study was covered with bubbles and was very difficult to distinguish.

All polyp images missed by the current algorithm were white polyps (upper left, Peutz-Jeghers syndrome; upper middle; Peutz-Jeghers syndrome; upper right, FAP). All of the malignant lymphoma images missed by the developed algorithm in this study were follicular lymphomas with white-lymph follicle-like bumps (middle left, follicular lymphoma; middle center, follicular lymphoma; middle right, follicular lymphoma). In contrast, the polyp training data were red polyps with pyogenic granuloma. The tumor image in the training data used in this study was covered with bubbles and was very difficult to distinguish.

## 4. Discussion

We developed a CNN algorithm to detect a variety of clinically significant lesions on images obtained by SBCE. Since the training dataset included images with abnormal findings that contributed to diagnosis, the CNN algorithm developed in the current study realized a high specificity and a relatively small number of detected images.

Previous reports have showed that lesion-specific algorithms have high sensitivity and specificity for angioectasia (approximately 98%–100%) [7,8], erosion (88.2%), and ulceration (90.9%) [10]. A meta-analysis showed that the pooled sensitivity and specificity for the detection of ulceration are 95% (95% confidence interval [CI], 89–98) and 94% (95% CI, 90–96), respectively [18]. Thus, CNN algorithms show high levels of diagnostic performance for angioectasia and erosion and ulceration in SBCE. In contrast, the lesion-detection performance of a previous CNN algorithm for polyps is relatively low (sensitivity 90.7%, specificity 79.8%) [19]. Similarly, our CNN algorithm developed to detect various lesions had a relatively low sensitivity for polyps (83.3%), although the overall sensitivity was relatively high (93.4%). The CNN algorithm developed in the current study also showed a lower sensitivity for lymphoma than for the other findings. After reviewing the images of neoplastic lesions in the training dataset, we assumed that the reason that our CNN algorithm had a decreased sensitivity for polyps and lymphoma was because the training dataset did not include images with similar characteristics to the false-negative images (Figure 5). In SBCE, the prevalences of polyps and lymphoma are relatively low; however, there is a variety of abnormalities such as vascular and neoplastic lesions in the small bowel. Hence, for use in clinical practice, it is important to develop an algorithm that can effectively detect polyps and lymphoma, with increased sensitivity for detecting all small-bowel lesions. To improve the CNN algorithm developed in the present study, there is a need for additional training data including cases with polyps and lymphoma.

The present results show that high specificity can be achieved by appropriately selecting training data; however, we consider that further improvements can be made in the future by examining the false-positive images (examples of which are shown in Figure 3). The main findings incorrectly judged by the CNN algorithm (i.e., the findings seen in the false-positive images) were the assessment of white debris as an ulcer, a normal blood vessel as angiodysplasia, and a dark part of the surface depression as angiodysplasia (Figure 4). If the algorithm results in many false-positive images (e.g., normal vessels identified as abnormal findings), this may greatly increase the reading time because inexperienced physicians may need to spend a lot of time determining whether such findings are normal or abnormal. It is necessary to contrive ways to further improve the specificity of the algorithm, while maintaining the high sensitivity; this may be achieved by training the CNN algorithm with normal images rather than only adjusting the training dataset of abnormal findings, as this could cause a decrease in sensitivity.

The conventional-reading method takes around 30 to 75 min to observe all the images taken in the small bowel [3,20,21]. The CNN algorithm developed in the present study reduced the number of small-bowel images requiring reading to 7.8%, excluding cases with massive bleeding. It is not easy to estimate the reading time directly based on the number of reading images because the reading time varies depending on the experience of the reader and the diseases of the patient being examined. However, if we assume that the number of detected images is substitutable for the number of images requiring reading in the small bowel, the reading could potentially be completed within 10 min with the CNN algorithm. Two comparative studies demonstrated that the reading time of SBCE is around 3 to 5 min using a CNN algorithm in the clinical setting [12,14]. In daily practice, a reduction in reading time will enable a quick diagnosis and notification of the results to the patient, which is especially important for patients requiring urgent treatment. From this perspective, it is very important to prove the effectiveness of the CNN algorithm in the actual clinical setting. Therefore, it is necessary to demonstrate the effect of the reduced reading time by conducting further study to compare the detection rates between the conventional reading method and the reading using the CNN algorithm.

## 5. Conclusions

In the present study, we developed a lesion-detection algorithm with a high specificity and a reduced number of detected images, which may contribute to a shortened reading time. By conducting appropriate extra training with neoplastic lesions, it is expected that the proposed algorithm can be applied to a SBCE-reading-support system to assist in detecting images of lesions in the future.

## Figures and Tables

**Table 1:** Characteristics of the validation dataset.

Number of cases		35
Number of abnormal findings		271
Age, mean ± SD		69.3 ± 14.3
Gender male/female		22/13
Indication		
	OGIB	20
	Anemia	5
	Abdominal pain	2
	Lymphoma	3
	Polyp	2
	FAP	1
	Other	2
Abnormal findings		
	Bleeding	30
	Angiodysplasia	46
	Ulcer	110
	Erosion	43
	Polyp	12
	Others	4
	Lymphoma	26

OGIB; obscure gastrointestinal bleeding, FAP; familial adenomatous polyposis.

**Table 2:** Performance of the CNN algorithm.

Sensitivity, %		93.4
	Bleeding	100.0
	Angiodysplasia	100.0
	Erosion	93.0
	Ulcer	92.7
	Polyp	83.3
	Lymphoma	57.7
	Others	100.0
Specificity, %		97.8
Number of detected images		7514
Number of detected images excluding massive bleeding cases		3576
